# Blister-like Cerebral Aneurysm after Endovascular Catheterization: A Case Report and Literature Review

**DOI:** 10.7759/cureus.39674

**Published:** 2023-05-29

**Authors:** Ebtesam Abdulla, Krishna Das, Harleen Luther, Andrew Amuah Wireko

**Affiliations:** 1 Neurosurgery, Salmaniya Medical Complex, Manama, BHR; 2 Medicine, Harvard T.H. Chan School of Public Health, Boston, USA

**Keywords:** rapture, endovascular, subarachnoid haemorrhage, blister-like, aneurysm

## Abstract

Endovascular procedures have become a mainstay in the treatment of neurovascular pathologies like arteriovenous malformations and aneurysms. Catheter-induced blister-like aneurysms (BBAs) have not been described so far in the neurosurgical literature. The authors report a rare case of a possible, catheter-induced (iatrogenic) BBA of the supra-ventral wall of the internal carotid artery (ICA) post-endovascular coiling for posterior communicating artery (PComA) aneurysm and bring the rapid progression of BBA and the grade prognosis. A 46-year-old female presented with convulsions. Imaging studies showed diffuse subarachnoid haemorrhage (SAH) and a right saccular PComA aneurysm. Endovascular coiling of the aneurysm was performed, and it was uneventful. The patient had a good outcome (modified Rankin Scale of 1) with no neurological deficits and was discharged home on day five. However, on day nine after the first ictus, she experienced a severe headache at home and was rushed to the emergency room where she collapsed. A cranial computed tomography scan showed intracerebral haemorrhage with intraventricular extension and SAH. A cerebral angiogram showed a BBA of the supra-ventral wall of the ICA. A BBA needs to be considered as a complication of an endovascular procedure that may result in rapid neurological deterioration post-coiling due to rupture. The report also illustrates the rapid and catastrophic presentation of BBA.

## Introduction

Blood blister-like aneurysms (BBAs) are microaneurysms that are less than 3 mm in diameter and compromise 1-2% of intracranial aneurysms in adults [1]. They are categorized by half-dome projections concerning the non-branching internal carotid artery (ICA) as anteromedial (65%), anterior (12.5%), anterolateral (12.5%), medial (5%), lateral (2.5%), and posteromedial (2.5%)[1-3]. Rarely, BBAs can be found on the anterior cerebral artery (ACA) (A1 segment), middle cerebral artery (MCA) (M1 segment), and vertebral artery (V4 segment) [4,5]. BBAs pose a surgical and endovascular challenge due to their fragility and broad-base appearance, making their occlusion using direct clipping or Guglielmi detachable coils difficult [2,3,6,7-9]. Excessive blood pressure, high cholesterol, and aberrant collagen synthesis are recognised patient-related factors that have been demonstrated to enhance the risk of BBAs formation and subsequent rupture [1]. However, there is no reliable way to identify individuals who are at risk for the formation of BBAs. A possibility of iatrogenic vascular injury secondary to endovascular coiling has not been recognized yet as a risk factor for BBA formation. Thus, with a review of the literature on this clinical entity, we report a case of a potentially ruptured, catheter-induced (iatrogenic) BBA of the supra-ventral wall of ICA post-endovascular coiling for posterior communicating artery (PComA) aneurysm.

## Case presentation

**Figure 1:**
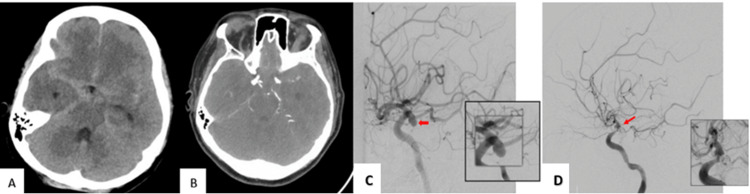
(A) Non-contrast computed tomography scan of the brain showed diffuse subarachnoid haemorrhage; (B) CT angiogram and (C) catheter angiogram showed the right posterior communicating artery aneurysm; (D) Post-embolization angiogram showed protection of the dome of the aneurysm

An otherwise healthy 46-year-old female presented with a new onset of convulsions and an altered level of consciousness (Glasgow Coma Score (GCS) 8/15). The patient had no prior history of traumatic head injury, fever, heart disease, or intravenous drug use. Non-contrast computed tomography (CT) scan of the brain showed diffuse, thick subarachnoid haemorrhage (SAH) (Fischer Grade 3/4) (Figure 1A).

**Figure 2:**
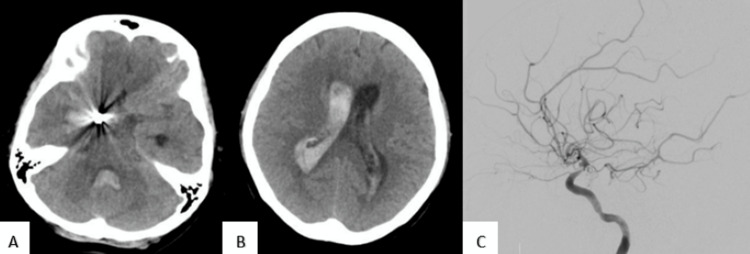
Non-contrast computed tomography brain showed right temporal intracerebral haemorrhage at the site of the coiled aneurysm (A) with intraventricular extension (B); Catheter angiogram showed a blood blister-like aneurysm arising from the dorsal wall of internal carotid artery (C)

Both CT arteriogram (CTA) (Figure 1B) and right carotid arteriogram (Figure 1C) revealed a 6.5×6 mm (depth, neck), wide-neck, lobulated, saccular aneurysm arising from the PComA segment of ICA, and fetal posterior cerebral artery. Endovascular coiling was performed to secure the aneurysm. The patient achieved a good outcome, a modified Rankin scale of 1 with no neurological deficits, and she was discharged on day five after the ictus. Post-embolization angiogram (Figure 1D) showed that the aneurysm was well coiled and the PComA was patent. However, on day nine post ictus, she presented with a severe headache and collapsed in the emergency room (GCS 4/15). The patient was intubated, and her vitals were normal. Non-contrast CT brain (Figure 2A, 2B) showed intracerebral haemorrhage adjacent to the coiled aneurysm with intraventricular extension causing ventriculomegaly and SAH.

An emergency external ventricular drain insertion was completed, and the patient was taken up for four cerebral vessel angiograms. The right carotid angiogram (Figure 2C) revealed a 1.5×2.5 mm (depth, neck) BBA arising from the supra-ventral wall of the ICA. The adjacent ICA, ACA, and MCA showed moderate to severe vasospasm that partially responded to arterial injection of verapamil. There was no flow in the coiled PComA aneurysm. A diagnosis of a ruptured BBA seen in the second angiogram was considered and the arrangement was made for treatment by an endovascular flow diverter. Unfortunately, the next day, the patient deteriorated and expired.

## Discussion

BBAs are uncommon lesions accounting for less than 2% of all intracranial aneurysms and 0.9-6.6% of ICA lesions [1]. Historically, in 1984, Yasargil and colleagues reported three BBAs among 319 carotid lesions [10], and in 1986, Nakagawa and colleagues reported eight BBAs among 460 intracranial aneurysms [6].

The anatomical distinction of BBAs is that the aneurysmal outpouching is covered only by a thin layer of tunica adventitia, which is prognostically significant given the high risk of rupture, rapid deterioration, and aggressive clinical course. Based on Gonzalez and colleagues' review, most BBAs cases present with a World Federation of Neurosurgical Societies (WFNS) grade > 3 (68%) [11]. Being initially microaneurysms, they can evolve into a saccular type within days of presentation which ultimately re-rupture with potentially fatal outcomes [7].

BBAs may occur slightly more often in young female patients with a predilection to the right side [12,13]. Based on Abe and colleagues’ series, the mean age at presentation was 56 years [14], in contrast to Park and colleagues' series, where the mean age at presentation was 35.4 years [15]. Risk factors include high systemic blood pressure, high atherosclerosis, and collagen vascular disease [1]. Although many of these factors are correlated with rupture, causality remains to be established. In our patient, the CTA and digital subtraction angiogram (DSA) did not show any abnormality in the vessel wall except for the PComA aneurysm. When the patient presented post-ictus on day nine, DSA was repeated, which demonstrated an aneurysmal outpouching on the supra-ventral wall of ICA, very near the PComA aneurysm. Given that the first angiogram did not exhibit any suspicious lesion in the said area, we felt that the BBA, which developed after the first procedure, was secondary to injury induced during the coiling of the PComA aneurysm. The sudden ballooning of a dormant blister aneurysm is also postulated as the second review on the angiogram done a few days earlier showed an ectatic vessel. Thus, suspicions of a BBA after endovascular catheterization should be considered as a potential aetiology for such clinical presentation. Joo et al. described a case of BBA that was at the right ICA bifurcation's dorsal wall. Repeated attempts to clip the BBA were unsuccessful because of its thin and fragile wall, small size, and wide neck, which caused a laceration, substantial bleeding, and hemodynamic instability. The authors stressed how crucial it is for anaesthesiologists to be prepared for the likelihood that saccular aneurysms may be identified as BBAs and that there will be considerable bleeding from an iatrogenic rupture [16,17]. Moreover, given the high risk of intraoperative rupture and parent vascular injury, microsurgical therapies of BBAs have historically been difficult and associated with poor outcomes [18,19]. Many complications have also resulted after conventional coiling or stent-assisted coiling (SAC) of a ruptured BBA, including intraoperative aneurysm rupture due to the weak non-collagenous aneurysm wall and coil embolization due to the broad neck of the aneurysm [20]. Although very uncommon with minimal elucidation in the current literature, this case report reflects the rarity of BBA after endovascular catheterization and the probability of said neurosurgical sequelae.

Treatment strategies available for BBAs involve endovascular (coils or stents) and microsurgical (clipping, wrapping, trapping with or without bypass, or angioplasty) options with no consensus on the ideal therapeutic plan [2,3,6,7]. Except for the newly introduced flow diverters, the efficacy of the treatment options is equivocal [3,16]. Currently, flow diversion is the most practical treatment approach with a high occlusion and low retreatment rate [16]. However, the treatment decision should be made on a case-by-case basis. Neurosurgeons and interventional radiologists should consider all available alternatives, both surgical and endovascular, to maximize the chances of a good outcome [1,2,16].

## Conclusions

BBAs are rare and usually present in severe clinical conditions. A sudden deterioration post endovascular coiling in the absence of trauma should raise suspicion for a potential BBA rapture. Given their rapid development and progression, BBAs necessitate urgent intervention to save the patient’s life.
